# Diagnosis of rare diseases under focus: impacts for Canadian patients

**DOI:** 10.1007/s12687-017-0320-x

**Published:** 2017-07-21

**Authors:** Daphne Esquivel-Sada, Minh Thu Nguyen

**Affiliations:** Centre of Genomics and Policy, Faculty of Medicine, Department of Human Genetics, McGill University, 740 Dr. Penfield Ave., Montreal, QC H3A 0G1 Canada

**Keywords:** Rare disease community, Genetics, Patients’ experiences, Diagnosis impacts, Qualitative analysis

## Abstract

This paper presents an in-depth qualitative analysis of the impact of diagnosis on the lives of rare disease (RD) patients. While diagnosis may be described as a watershed step for RD patients, no extensive account of non-medical outcomes following a RD diagnosis exists within the literature. This study aims to fill this knowledge gap through an analysis of the impact of diagnosis on the lives of RD patients according to their personal experiences. Qualitative research was conducted in three provinces across Canada, with a total of 23 participants, both adult and parents of children with RD, diagnosed and not yet diagnosed. A thematic approach guided the analysis of the transcripts. The results reveal that the impacts of a RD diagnosis for both adults and paediatric patients are multifold, ranging from social to personal and medical impacts (including cases where etiological treatments for the diseases are non-existent). Furthermore, the results shed light on distinct factors that affect the scope of impacts of a diagnosis.

## Introduction

In spite of the growing public awareness of the medical and social issues surrounding rare diseases (RDs) over the last two decades (Bavisetty et al. 2013; Schieppati et al. 2008), inequality in the distribution of heath care and ancillary support services remains a reality for RD patients (Barrera and Galindo 2010; Elliott and Zurynski 2015; Khangura et al. 2016; Kole and Faurisson 2010). The burden of a RD is often also exacerbated due to disease misdiagnosis and unknown aetiology. Although diagnosis may be described as a watershed step for RD patients confronted with a myriad of challenges (e.g. lack of scientific knowledge and information, inadequate comprehensive health care, and expensive drug treatments), it may represent an odyssey for these patients, as it may take up to decades for an accurate diagnosis (Alonso et al. 2011; Engel et al. 2013; EURORDIS 2009).

Considering that the majority of RDs are said to be genetic-based (Barrera and Galindo 2010; Schieppati et al. 2008), the advancements and accessibility of next-generation sequencing (NGS) techniques are expected to help address diagnostic challenges faced by the RD community by facilitating both the ability to establish a diagnosis and pinpoint new RD-causing genes (Madrigal et al. 2014; Pierson et al. 2012; Prada et al. 2014; Reuter et al. 2015).^1^ Although the potential of new genetic diagnostic technologies to allow for better management of diseases is promising, their impact on clinical practice is still limited (Basho et al. 2015; Buchanan et al. 2013; Reuter et al. 2015).

Conflated with current health care resource allocation dilemmas, most test evaluations focus on clinical outcomes, thereby implying the necessity of having available and effective preventive or therapeutic options (Buchanan et al. 2013; Bunnik et al. 2014; Foster et al. 2009).^2^ Such clinically driven criterion raises important issues regarding RD diagnosis, if one considers the particular situation and experiences of the RD community (Alonso et al. 2011), and that numerous RDs currently lack treatment (EURORDIS 2009; Schieppati et al. 2008). Therefore, the “right to a diagnosis” remains the object of significant debate in the RD community. While most RD patients continue to search for a diagnosis, regardless if a treatment exists, some health care providers feel that it is unethical to “announce to a patient the diagnosis of a disease for which nothing can be done” (Kole and Faurisson 2010:247). The issue is further exacerbated when considering public health care systems that face limited budget constraints and must balance individual and collective rights and interests.

How should such complexity be addressed? Despite calls for research “[…] to identify and quantify the nature of benefits of genetic testing [other than medical] from the perspective of the individuals to whom testing is offered” (Grosse et al. 2008:652), a literature gap persists regarding empirical accounts from the perspective of patients (Bossuyt and McCaffery 2009; Segal 2012). In addition, most studies that have been conducted on this topic have adopted a quantitative approach (Regier et al. 2009). As for the qualitative studies that have been reported, they remain either too broad in scope (i.e. providing analyses from a common-disease perspective) or are limited to specific RDs (Behan et al. 2016).

Our study aims to help fill this knowledge gap by examining specific challenges and realities of the RD community through the experiences of RD patients. Through focus group (FG) discussions with RD patients, our objective is to provide a comprehensive and in-depth empirical account of the impact of diagnosis from the perspectives of these patients. In doing so, our goal is to draw a more comprehensive and fine-grained portrait of potential outcomes of diagnosis for RD patients. The questions guiding our inquiry are what are the experiences and perspectives of RD patients with respect to diagnosis across Canada? What particular dimensions (social, subjective, medical, etc.) frame their experiences of obtaining a diagnosis? What are the different impacts of a diagnosis, both positive and negative, on the lives of RD patients according to their experiences? In doing so, our study also brings to light factors that limit the impact of diagnosis for RD patients.

Grasping the role that diagnosis may play in the lives of patients with a RD, the array of impacts that it may yield on their lives along with their specific needs and challenges seems necessary for the eventual implementation of NGS and other diagnostic tools for RD. That being said, this study is not framed to answer public health policy issues relating to genetic testing. Rather, our study provides a comprehensive empirical analysis of the impacts of receiving (or not) a diagnosis for RD patients. In that sense, we hope that the outcomes of our study will inform future deliberations and evaluations for the clinical implementation of diagnostic genetic/genomic tests responsive to the RD community challenges.^3^

## Methods

In order to get a thorough account of the impact(s) of diagnosis on RD patients’ lives, we conducted in-depth FG discussions with adult patients and parents of children with RD, diagnosed or not yet diagnosed. Our chief goal was to grasp the range of impacts of RD diagnoses through the embodied knowledge of participants having first-hand experiences with RD diagnostic processes and their outcomes (Patton 2002; Starks and Trinidad 2007). We conducted six FGs in three provinces across Canada (five in the province of Québec, one in Calgary, and one in Ottawa) and one individual interview (II). FGs were conducted in English or in French depending on the participants’ preferences. The provinces were chosen for convenience purposes as they were sites where C4R investigators are located. All meetings were held in person.

FG methods were favoured to elicit the range and depth of perspectives on RD diagnoses. They allowed taking into consideration the exchange of ideas, experiences, and perceptions amongst participants, while illuminating their contrasts (Patton 2002). Furthermore, a productive aspect of the FG method with regard to patient communities is that because it relies on social interaction, it is thought of as a tool for the empowerment of such populations (Lehoux et al. 2006).

Participants were recruited via genetic clinics in several hospitals, free online advertisement websites (Craigslist and Kijiji), and genetic RD Canadian patient associations (approximately 40 associations), which published our recruitment ads in newsletters and websites. The sampling strategy was twofold in order to distinguish the divergences and similarities of views and experiences between subgroups. It aimed at having both adults and parents of children with RD, in order to account for the particular ethical and legal issues that frame genetic testing technologies in the adult and paediatric populations^4^ (Geelen et al. 2011). The sampling strategy also sought to include both diagnosed and undiagnosed patients; the experiences of the former allow us to grasp the array of impacts of a RD diagnosis, while those of the latter shed light on how a lack of diagnosis may impact their lives. A total of 23 participants were recruited, and participants were given $100 CAD to compensate for costs related to travel, parking, and child care services.^5^

The socio-economic profiles and the ages of the participants were diverse. However, only one participant was male. For the 23 participants (i.e. both adult patients and parents of children with RD), we had a total of 25 patient cases discussed during the FGs and the interview.^6^ Therefore, Table 1 differentiates between participant information and patient information; the former refers to all adults who participated in the discussions, while the latter comprises both the adult participants with RD and the children with RD whose parents were participants at the FG. As Table 1 indicates, out of the 25 patients, 23 have received a diagnosis,^7^ and of those, 20 were genetic-based RDs. In total, 19 different RDs have been diagnosed. The large majority of these are inherited genetic mutations (with the exception of one non-genetic based RD, one RD related to de novo mutations, and two RDs whose genetic base is still unknown).^8^

**Table 1:** Participants socio-demographics and patient information

Discussions	Participant information (data for all adult patients and parents who participated in FG)			Patient information (data for all adult and children RD patients)	
	Gender	Age	Education	Received RD dx	Genetic based RD
II-F	Female	29	University	Yes	Yes
FG-A-E	Female	36	University	Yes	Yes
	Female	51	Masters	Yes	Unknown
	Female	44	University	Yes	No
FG-A-F	Female	35	University	Yes	Yes
	Female	52	University	Yes	Yes
	Female	42	High school	Yes	Yes
FG-MIX-F	Female P	35	College	No	NA
	Female P	37	College	Yes	Yes
	Female P	27	High school	Yes	Yes
	Female A	33	High school	Yes	Yes
FG-P-F	Female	44	College	Yes	Unknown
				Yes	Yes
	Female	48	University	Yes	Yes
	Male^a^	58	PhD	Yes	Yes
				Yes	Yes
	Female^a^	59	University	Same as participant above	Same as participant above
FG-P-E1	Female	53	College	Yes	Yes
				Yes	Yes
	Female	27	College	No	NA
	Female	52	University	Yes	Yes
	Female	56	University	Yes	Yes
	Female	34	College	Yes	Yes
FG-P-E2	Female	54	University	Yes	Yes
	Female	41	University	Yes	Yes
	Female	57	Not available	Yes	Yes
Total	Participants = 23 (8 adults, 15 parents)			Patients cases = 25	

*II* individual adult interview, *P* parent of paediatric patient, *A* adult patient, *F* French, *E* English, *MIX* parent and adult patient FG, *NA* not applicable

^a^Indicates a couple whose adult children are affected by the same disease as the mother.

The FGs were held in conveniently located meeting rooms (community centres, hospitals, universities, and hotels). Each FG lasted between 2 and 2.5 h and consisted of between three and five participants. Prior to the FG, participants signed consent forms and completed a socio-demographic questionnaire. At least two researchers were present at each FG, one assuming the leading moderator’s role and the other assisting in logistics and note taking. The discussion and the interactions between the participants were moderated with the aid of a guide adapted for each subgroup of participants, which consisted of three central issues: (1) description of disease and diagnosis experience, (2) general perceptions and experiences regarding genetic testing, and (3) specific views on NGS. The questions were developed based on common RD community challenges, social and ethical issues relating to genetic testing in general, and NGS issues. All FGs were audio recorded and transcribed, with prior permission from the participants.

Transcripts were imported onto the NVivo 8.0 software. The content of the first five discussions was coded independently by two analysts following the principles of thematic analysis (Attride-Stirling 2001; Braun and Clarke 2006) in order to obtain exhaustive inductive codes. After discussion, relevant codes for the research questions were then organized by the main analyst into three thematic categories: impacts of diagnosis, upstream challenges to obtaining a diagnostic genetic test, and views on NGS.

The scope of this paper focuses on the range of impacts that a diagnosis may have on the lives of RD patients and factors undermining such impacts. Our analytical framework approach seeks to yield a thematic description of the elements raised during the FGs and the interview. Our analysis presents participants’ perspectives in accordance with the complexity of the experiences that they described (Patton 2002; Starks and Trinidad 2007).^9^ When relevant, verbal exchanges and interplay between and amongst participants were illustrated to conform to the interactive nature of FGs (Hydn and Blow 2003).

## Results

The analysis of the discussions reveals that the impacts of receiving a diagnosis for RD patients are multifold, ranging from direct and indirect medical impacts to social and personal impacts. Such impacts can be positive and negative as well as complex, to the extent that their scope depends upon different factors. Three major themes, with several subcategories of outcomes, arose from the accounts of experiences (Table 2).

**Table 2:** Summary of impacts of a diagnosis for RD patients

Medical impacts
• Medical intervention: a diagnosis may allow receiving proper treatment while avoiding “mistreatments”
• Health care: a diagnosis tends to ease access to health care but may also present new obstacles for patients
• Age-based asymmetry between paediatric and adult patients: access to medical and support services tends to vary for paediatric patients compared to those diagnosed as adults
• Indirect health-related impacts: a diagnosis may result in lifestyle changes for the patient
Social impacts
• A diagnosis allows access to ancillary public services (social welfare, subsidies for special health care and needs)
• A diagnosis can provide a means to connect with specific RD support groups
•A diagnosis may entail insurance discrimination and stigmatization
Personal impacts
•A diagnosis allows patient empowerment, self-confidence, and a gain of respect vis-à-vis medical professionals
•A diagnosis may offer relevant information for life planning and reproductive decision-making
• A diagnosis (or the medical acknowledgement) of a genetic condition may spur family conflicts

### Medical impacts

By “medical impacts”, we are referring to changes in medical care, treatment, and coordination of care when a person obtains a diagnosis. According to the participants, obtaining a RD diagnosis affects four main dimensions related to medical care: (i) medical interventions, (ii) access to health care, (iii) age-based asymmetry between paediatric and adult patients in the quality of medical care, and (iv) indirect health-related impacts. The medical outcomes reported by the participants are not always straightforward, but take place in a complex interplay of factors.

#### Medical intervention

An accurate diagnosis may lead to good disease prognosis, long-term treatment, and medical care. Yet, this kind of direct medical impact is exceptional for our participants since most RDs do not have treatment options. Regardless, even where no proper drug therapy exists, diagnosed patients may still receive symptom-alleviating treatments, as explains this participant:

“[…] once I had the diagnosis, then the doctors took me seriously, even those who had no idea of what it meant. But because I had a diagnosis, which seemed serious, I saw an internist. At once she gave me medication […] to increase my blood volume. Within three days, my quality of life increased by 80%.”(A-F).

Regardless of the lack of treatment, a diagnosis may also prevent patients from receiving unnecessary health care (e.g. “overtreatment” or “mistreatments”). Undergoing unnecessary medical tests was a common complaint amongst our participants, as stated by a mother whose child’s diagnosis took 19 years to be obtained:

“[Because it took us nineteen years to obtain my child’s diagnosis, now I know that] another thing that would have saved my child and our family a lot of grief and a lot of pain would have been knowing that we didn’t need to do all those biopsies, we didn’t need to do all those tests. She didn’t need to have those surgeries. […] It would have saved her so much pain.” (P-E1).

In the same vein, visits to the emergency room have often been pointed out as a source of unnecessary burden atnd mistreatment (e.g. prescription of antibiotics for chronic diseases). Yet, seeking emergency services appears to be the only available option for RD patients who have not yet received a clear diagnosis and have no physician following them:

“[Because of face pain] I have been to the emergency three times waiting four-five hours to be prescribed needless antibiotics. They eventually discovered [years later] it was actually an inflamed nerve [rather than sinus infection].” (II-F).

#### Access to health care

Even though the Canadian public health care system ensures free health services, many participants highlighted the difficulties that they encountered prior to obtaining a diagnosis. For them, receiving a diagnosis essentially represented an “entry ticket” to the health care system, enabling them to receive proper follow-up and access to different medical specialists and services:

“[The access to health services] has snowballed since my diagnosis. It became much more efficient.” (II-F).

The coordination of care was also brought up by many participants as an important challenge for both parents and adults. Given the often composite nature of RD, such patients require multiple specialists and struggle to coordinate the myriad of necessary medical appointments. Once diagnosed, many RD patients explain that they gain access to a physician, such as a paediatrician or an internist, who will play the lead in coordinating the required medical care and services:

“[…] I have an internist. He’s kind of the conductor of every other specialist […]. Prior to that, it was always very burdensome […]. I had to remember by myself the frequency at which each specialist wanted to see me. I have twelve specialists […]. Then most of the time I didn’t follow up properly. […] So now, [I have] my internist managing all that […].” (II-F).

Interestingly, depending on the disease in question, some patients declare that their diagnosis actually hampered their medical care. Below, a participant who suffers from a connective tissue disorder shares her experience:

“[…] I’m not able to find an orthopaedist to follow up my case, to save my life. I was told that according to the Medical College, they are not allowed to touch me. I have verified this information [with the RD association and they] answered it is not true. However, it is about the tenth or eleventh orthopaedist that refuses me.” (A-F). 10

#### Age-based asymmetry between paediatric and adult patients

Throughout the discussions, several participants exchanged views on how changes in terms of care and follow-up after a diagnosis tend to be different, depending on whether you are a child or an adult with a RD. Adult patients who had been treated since childhood, and gone through what is referred to as an “age transition” (i.e. becoming major), have pointed out striking differences in medical follow-up and coordination of care. The following adult patient, who has been severely sick since childhood, experienced this contrasting transition into adulthood:

“[...] in the adult system, it’s so different than when you’re in the children’s network… […] because I had something that was considered at the time very rare, I was a bit of a star patient, and so I got a lot of attention, which I found shockingly absent when I eventually had to get services as an adult […]. When I started to have problems with my spine [as an adult], and tried to get the orthopaedic surgeon to talk to the neurosurgeon or the neurologist, they weren’t collaborating at all […]. Whereas […] when it was the Children’s Hospital, they would have three different […] teams from three different hospitals meeting to talk about me, whereas [as an adult] I couldn’t get people in the same… […] hospital to talk. And I ended up having, stupidly, surgery…” (A-E).

This excerpt brings to light the lack of interaction and communication amongst specialists caring for adult patients. This asymmetry in care is also felt when paediatric patients reach the age of majority since most patients are placed on general waiting lists when transitioning into the “adult” health care system. The “new adult” is no longer followed by paediatric specialists and therefore must find new health care professionals. This can be extremely challenging in certain cases. Below, a parent expresses the range of new responsibilities that an adult child may acquire:

“[...] as soon as my child had the diagnosis at the children’s hospital, they knew where to send her, and she had a myriad of appointments. And then she turned 18. […] then all of a sudden I had to be the organizer, as well as the knowledge base, as well as […] had to fight for each and every one of the monitoring visits […]. And each single one of those [specialists], I had to get a referral from [the] family doctor every single time to go for the yearly check-up […].” (P-E1).

Proper access to care appears be all the more difficult for patients diagnosed in adulthood. Here, we have an exchange between parents of infants and a couple whose children were diagnosed in adulthood. They discuss the disparate treatment that their children received because of their age:

“P1^11^ (minor child): we see the paediatrician once a year at the [children’s hospital]. The neurologist twice. The neuro-ophtalmo twice. Across those, hearing tests also […].

P2 (adult child): [In our case, one of our adult children is still waiting for a family doctor available to follow her], and the other waited a long time before seeing one […].

P3 (adult child): That’s it. We don’t have the same experience as you two parents [of minors] because we learned that our children suffered from this disorder [when] they were in their twenties […].” (P-F).

In light of the different challenges faced by adult and paediatric patients within the health care system, it is less surprising that some adult participants claimed that receiving an official RD diagnosis has not led to any tangible change in their medical care:

“I have to say that getting a diagnosis did not open up any services for me. I just recently, after seven years, found a neurogenic bladder specialist after searching for so long.” (A-E).

“[After having receiving the diagnosis], we asked [the geneticist]: now what is this information good for? What can we do with it? He started laughing and said, ‘No, there is nothing to do about it. You must bear it. A research group will eventually start. We will contact you.’ We are still waiting. It’s been five years.” (P-F)

#### Indirect health-related impacts

In addition to directly impacting health care for RD patients, a diagnosis may entail more diffuse life-changing health outcomes, such as effects on lifestyle. According to some participants, depending on their disease, an earlier diagnosis could have prevented the deterioration of their condition.

A patient with a deteriorating disease, and whose condition is worsened by any type of tissue trauma (including muscle stretching), explains below how an early diagnosis could have changed her lifestyle:

“In hindsight. If [after a diagnosis] someone had told me the first times I had joint dislocations: […] stop doing contact sports […], I’d be much less affected today than I am. [Wheel chair, walker, orthotic device] are stuff that could have been avoided [at my age], had I knew [my diagnosis] younger [than 31 years old].” (A-F).

Finally, insofar as a diagnosis implies genetic information, the indirect medical impact may not relate to the patient’s individual health but to their family members. A mother explains here how the lack of a diagnosis for her sick child left her feeling blind to her own health condition and those of her other children:

“[Because we don’t have a diagnosis for my sick child] we don’t know a whole lot of other stuff [beyond the choice of medical intervention for my sick child]: […] we don’t know whether or not our [other child, who is asymptomatic so far] is also affected. […] I also have [similar symptoms].” (P-E1).

### Social impacts

According to the participants, obtaining a diagnosis can lead to important social outcomes as well. These range from eligibility to ancillary and community services, accessing support groups, and insurance discrimination.

#### Access to ancillary services

In Canada, being given an official diagnosis is imperative for one to be eligible for various social and ancillary services (e.g. social welfare for families caring for sick individuals). Such services can offer financial help for basic needs, such as special food and medical equipment, which can often have exorbitant costs. In the following excerpts, two mothers, whose children are still awaiting a diagnosis, explain the following:

“The great big Holy Grail of all things in [our province] for getting access to everything is [a program for support for children with disabilities]. In order to qualify for [it] you have to have a diagnosis, and [my child] does not have a diagnosis. […] we pay $3,000 a month for formula, [and] thousands of dollars a month for medical supplies for her hospital stays […]. All of those things would be covered by [the program] if only she had a diagnosis. So, despite the fact that we’ve known that [my child] has all these [severe] problems, and despite the fact that she’s seen in 19 different clinics at the Children’s Hospital right now, she has not qualified for support because she does not have this miraculous diagnosis. Saying that you have […] a category of a disease [without] the specific genetic identifier of whatever it is, is enough for them to disqualify you.” (P-E1).

“[A diagnosis is important] to have subsidies. I’ve knocked on several doors. I was told: […] what do you have proving [your child] has whatever it is?” (MIX-F).

When it comes to parents whose children have obtained a diagnosis, their experiences are contrasting. A diagnosis entitles them to government support for fundamental needs as well as to special services:

“[With the diagnosis] you get the services, and also school support. She has an [educational assistant] that is like her bodyguard at school, to protect her from the other kids, and also now, […] they’re lifting her in and out of the stroller to bring her out to play. She can’t wipe herself when she goes to the bathroom […]. All these [support services] would’ve been really hard to get without a diagnosis.” (P-E2).

#### Access to patient support groups

Some patients, after having a diagnosis established, were able to associate themselves with different RD communities and patient support groups. Discovering other disease-like kin and realizing that one “is not isolated” are clearly seen as a source of psychological comfort. Yet, the benefits of patient support groups are not limited to psychological alleviation but can be remarkably composite. Because of her involvement with a particular RD community, one mother explained that she learned how to care for her toddler who suffers from a connective tissue disease to prevent traumas and gained access to a world specialist on the disease:

“P1: For us having the genetic diagnosis was awesome. It opened so many doors […]. First of all, we got the name [of the disease] and then we found our ‘family’ […]. The [x] community is an incredible community full of resources […]. It gave us access to this doctor [outside Canada] who is the world expert and has a research lab. The research lab runs off of funding of parents and families of children with [this disease].” (P-E2).

Support groups represent such an important element for RD patients that three of the participants had each created their own support group when none related to their disease existed.

#### Insurance discrimination

From a social perspective, a diagnosis may nevertheless entail severe negative impacts for RD patients, such as insurance discrimination. This issue was raised by participants and considered during various discussions. Although the Canadian health care system is publically funded, a RD diagnosis may prevent patients from obtaining life, travel, and disability insurance. The following excerpt illustrates the kind of secondary aftermaths that such injunction may entail:

“I stayed with my job because […] if I change now [after my child turned 18], she will come off [my insurance plan]. I will stay here ‘til I die because she’s covered. My husband, however, has changed [his job], and my child has, as soon as she turned 18, no coverage [on his side].” (P-E1).

### Personal impacts

The third major type of effect of a diagnosis on RD patients’ lives concerns the personal impacts. By these, we refer to all emotional-, cognitive-, psychological-, and family-related consequences of a diagnosis.

#### Empowerment, self-confidence, and respect

From the perspective of our participants, a diagnosis appears to be an empowering *tool*, enabling them to gain control and act proactively:

“[Finding out the diagnosis] is a moment of deliverance because we are able to put a name on it then change course, roll up our sleeves and say: ‘we will find solutions’ […]” (MIX-F).

Such ardent action is also spurred by a sense of self-confidence stemming from obtaining a diagnosis:

“P1: I wish I had known exactly what was wrong the day she was born if I could have, because then I could have been proactively just pursuing that, instead of fighting with people. I’d have been more direct and less questioning myself – because you do.

P2: Oh yes [laughs].” (P-E2).

Indeed, self-confidence appears to be paramount for patients with RDs. In this regard, it is important to recall the prediagnosed experiences discussed by many participants: prior to receiving an official diagnosis, their disease claims were often disavowed by physicians who would argue that the reported symptoms were psychosomatic, overstated, or provoked (e.g. patients suffering from a lack of attention). In the following excerpt, a mother recalls such underestimation or disregard for her child’s condition:

“[A specialist claiming that my child’s eye problems were only common and benign] said to me: ‘This is your first child?’ I said: ‘Yes.’ The end. He stopped investigating. It was my first child. I was a crazy mother. I was overstating the situation.” (MIX-F).

Some adult patients who have been sick since early childhood revealed during the FGs that the medical disavowal of their health condition went as far as making them doubt themselves. Such self-doubt may entail not only subjective suffering but health consequences as well, to the extent that it may deter patients from divulging new symptoms:

“For about seventeen years I heard that I was a girl creating problems […]. ‘She can’t have that many problems’, [they used to say]. So… I learned very young that all that was my fault. Thus, I also learned not to tell. When a new symptom came up… it had to become very, very serious to be worth telling.” (II-F).

Once the official diagnosis was provided, several participants, both in the adult and parent groups, emphasized the positive changes in their relationship with physicians. The diagnosis gave them a sense of respect when dealing with doctors, who had been downplaying and denying the medical evidence of their symptoms and suffering thus far. One patient, who obtained an accurate diagnosis after more than three decades, explains its importance, notwithstanding the lack of treatment for her life-threatening disease:

“[…] for me, it was mainly [to know my life expectancy] that I wanted to know [the diagnosis]. And also, because I’ve never been… I think the appropriate word here to be kind, it would be *respected* by the physicians. As I’m telling you, they’ve accused my parents of abusing me. The [X] hospital has refused to replace a dislocation if I didn’t make avowals that my husband hit me. And my husband didn’t hit me. […] For me, [receiving this diagnosis], basically, it’s just: ‘Ah ha! I was right!’ […] because you […] were told during a large part of your life that you were crazy, that the problem was between your two ears.” (A-F).

The diagnosis represents, overall, an official “validation” of their health problems. This was expressed in various ways by different participants. Most of the diagnosed participants reported how the diagnosis enabled them to finally be heard and be taken seriously by medical professionals:

“[After receiving a diagnosis] my general physician took me seriously and started helping me, making sure I got the specialists I needed.” (P-E2).

#### Life planning and reproductive decision-making

Both adult and parent participants stressed the importance of a diagnosis for life-planning purposes. A diagnosis provides insight into “what is coming ahead” in order to prepare oneself psychologically, to solve personal issues (e.g. resolving inheritance and family issues), and to make pragmatic decisions based on the prognosis (e.g. housing and debt resolutions). But the prime life-planning concern for the participants remains the possibility of making reproductive decisions based on the genetic information gained from a diagnosis.

In most of the discussions, the issue of reproductive decision-making was mentioned spontaneously and referred to in several contexts. Many participants were concerned with the transmission of genetic diseases to their offspring, as illustrated by this excerpt of a mother whose child is undiagnosed:

“[Knowing my child’s diagnosis] would [also] let me know whether or not we can have more children. […] We would like to have that [reproductive] choice.” (P-E1).

To the extent that a diagnosis reveals genetic information, the scope of interest for other family members was also often stressed, notably for siblings:

“P1: […] I think bigger [than having got the diagnosis for my sick child after nineteen years], for us right now it is for our other children. Because this is a [recessive genetic disorder] […], there’s potential for this to carry on with them and, […] so we feel really fortunate that they can now have the testing to know if they’re carriers or not.” (P-E1).

Moreover, for many participants, passing a genetic test is seen as a way to help relatives (e.g. nephews and nieces) with family planning decisions. They therefore regret that the tests are not available for healthy individuals in the Canadian public system.

Interestingly, while participants who raised reproductive implications were eager to obtain genetic information for reproductive decision-making, one couple with adult children was an exception. This couple sustained throughout the session that their surviving daughter deeply regretted having found out her diagnosis for an untreatable RD. As they explain below, the fact that her sibling passed away from their common conjunctive tissue RD has prompted doctors to associate her condition with a high-risk one, thus strongly discouraging her from becoming pregnant despite her desire to do so. According to these parents, doctors would be fearful of following a pregnant patient with such conditions given the foreseen medical complications^12^:

“Parent 1 (mother): She was the one who first had the insight about our disease […]. And she regrets it. She regrets having found that out. Because otherwise she wouldn’t be in the situation of [not being able to have children now]… […] She would have it and that’s it [just like me, her mother, who suffer from the same disease and] had two children and nothing [bad] happened. […] But she was told: ‘[…] you have very serious problems […]’. [Now] there are no more doctors willing to touch someone like that.

Parent 2 (father): In her case, she regrets it. […]. She wants to have children, the doctors don’t [want to follow her].” (P-F).

#### Family conflicts

During the discussions, conflicts amongst family members were rarely brought up explicitly as an “inconvenience” associated with receiving a RD diagnosis. Rather, this point was raised when participants were asked whether they would share results of diagnostic testing with other family members. Drawing on their past experiences, some participants discussed how the sharing of a diagnosis has provoked family conflicts and triggered a search throughout the family lineage to determine to whom the “faulty” inherited gene belonged:

“[…] when I shared [my child’s diagnosis with my family and more than ten nephews and nieces], because I was worried about them, it broke out a terrible family war. They launched bullets at each other. They did not know if this [disease] came from the side of my mother or the side of my father, from grandparents, etc., etc.” (A-F).

When asked whether they have experienced any drawbacks other than insurance discrimination after receiving a diagnosis, a few participants did respond that they had been the object of discrimination from family members. Here, two mothers point out how their families have been excluded:

“As soon as they [my extended family] found out about my child’s condition, it changed the dynamics completely. My mother won’t allow me to bring my child to her home [anymore]…” (P-E2).

“[My husband’s relatives] do not come to our place. No. They’ve never come to our place. The help I have, it’s all from my parents.”(MIX-F).

Whereas some have been almost excluded from family relations, with relatives cutting ties, other participants recalled that an official diagnosis does not necessarily persuade other family members that someone in the family has a genetic disease. Conditions were sometimes disclaimed by relatives, who would persistently call the family member with the disease a hypochondriac or blame that person for the genetic disease. The exchange below between three participants illustrates how common this issue can be:

“P1: I went to this [big family reunion with hundreds of first and second cousins] armed with this information [on the genetic diagnosis] to share. It absolutely exploded on me […]. [My aunts] turned on me […] saying, ‘How do you know? […] It must have been because you smoked marijuana in high school.’ […] the family turned on me. Instead of embracing the information, accepting it and pursuing the information to help themselves […]. I [couldn’t] imagine what opening Pandora’s Box could do. […]. In fact, I have not been back to a family reunion for more than three decades. […].

P2: Similar thing happened to us, too. […] My first cousin had two children with [symptoms]. When we got the diagnosis, I told them what it was and that I could have their geneticist talk to our geneticist. They absolutely cut all ties, and […] I have not seen them since then. […].

P3: I was going to say, actually… both what you’re saying ring a lot of bells. […] we gave this letter to everybody in our family from our doctor. We don’t have the specifics [genetic information of the disease], but we do know that there’s this general risk. And it’s funny. About a quarter of my family hasn’t talked to me since then. […] And interestingly enough, my […] sister who has a [child with similar symptoms] patently refuses to take him to a cardiologist because they don’t want to know. […] That same sister, over the course of the last year, tried to rally my family saying that I must have post-partum depression because I’m making up all these illnesses about my son.

P1: Oh, yeah. […] I had that one, too!

P3: […] But this suggests that there’s something wrong with us… […] that is a concern when talking about, perhaps, further genetic testing. That just means that certain relationships are going to be different.” (P-E1).

## Discussion

Our findings indicate that the medical, social, and personal impacts of a RD diagnosis for both adults and paediatric patients are significant, multifold, and ambivalent. Following the experiences of the participants, even in the absence of an etiological treatment for the RD in question, the medical outcomes may be cogent. Obtaining a diagnosis is a determining factor for proper medical and health care, which can include symptom-driven treatments, prevention strategies, and avoidance of unnecessary interventions and surgical procedures.

At the same time, our results reveal that the scope of medical impacts following a RD diagnosis is influenced by the patients’ age. To be sure, the issue relating to the challenging transition from childhood to adulthood as it relates to the care of chronic and RD (Molster et al. 2016; Murris-Espin et al. 2016; Rodger et al. 2012; Zurynski and Elliott 2013) is at play. Following the experiences of the participants, the Canadian health care system would benefit from building on local transition programs already in place for particular RDs (Dogba et al. 2014). However, the *asymmetry between adult and paediatric patients with RD* suggests that problems faced by adult patients go beyond the transition issue. While a positive change in medical follow-up and services was concordantly acknowledged amongst parents of diagnosed paediatric patients, many adults often expressed obstacles in obtaining follow-up with a general practitioner, long waiting lists for specialists, and physicians refraining from long-term follow-up. These patients tend to experience inadequate (notably, difficult access to specialists and lack of interaction between them) and uncoordinated medical care and services when compared to paediatric patients. Such asymmetry appears to be an offshoot of the structural differences between the paediatric and adult settings in the Canadian public health system.^13^ As such, this issue, which is also observed in other countries (Molster et al. 2016), would require further studies and reflections on the development of health care policies for the RD community, whose diseases are often chronic and composite.

The experiences categorized under the realm of social impacts reveal the paramount role that a diagnosis has in establishing relationships between RD patients and public institutions. Once diagnosed, RD patients become socially *visible* and *recognized*. Nonetheless, such visibility represents a double-edged sword, at least for certain patients. Obtaining an official diagnosis can signify eligibility and legitimacy vis-à-vis public institutions (e.g. access to social benefit programs), patient support associations, and a host of key community and secondary services (driven not only by public institutions, but also by philanthropic and civil society associations), which are diagnosis dependent. Yet, once diagnosed, patients increase their “visibility” vis-à-vis insurance companies and employers. Most of the participants shared their fears regarding the use of genetic test results to discriminate by denying patients access to insurance (Yaneva Deliverska 2011). However, in Canada, a Genetic Non-Discrimination Act came into force with Royal Assent in May 2017. Under this act, no one is obligated to undergo a genetic test or disclose the results of a genetic test to obtain services. Because the Canadian law is new, it remains to be seen how well the law is implemented to protect patient rights and what limitations and loopholes exist that may weaken its effectiveness.

The personal impacts stemming from receiving a RD diagnosis bring to light some of the micro-social outcomes of a diagnosis, notably on family relationships, reproductive decision-making, and personal life (emotional, cognitive, psychological). It is common to find in the literature a list of general genetic testing outcomes (not RD-specific literature) that are broad and abstract, such as effects on well-being and mood, as well as influences on future life planning (Bossuyt and McCaffery 2009; Payne et al. 2008). By drawing on the experiences of RD patients specifically, our analysis exposes the complexity, diversity, and, more importantly, the concreteness of possible “personal outcomes.”^14^ Notably, an official diagnosis allows for RD patients to gain self-assurance, respect, and legitimacy vis-à-vis health care providers, who in turn consider patients’ ailments more seriously, a result that supports previous research (Behan et al. 2016). In this sense, a diagnosis may be said to be a *transforming trigger*, encouraging positive relationships between patients and professionals and providing a basis for RD patients’ voices to be acknowledged. Without a diagnosis, most patients appear to have suffered from what scholars have called a “medical denial of the undiagnosed disease” (Kole and Faurisson 2010), having their narratives disregarded for various reasons (e.g. need for attention, overreacting, and an embellishment of symptoms). Such “psychologization of medically unexplained physical symptoms” by physicians (Atkins et al. 2013) might be associated with the common lack of knowledge and understanding of RD in the medical field, particularly amongst general practitioners (Anderson et al. 2013; Behan et al. 2016; Elliott and Zurynski 2015; Engel et al. 2013; Knight and Senior 2006).

On a more personal level, obtaining a diagnosis also allows RD patients to make pragmatic resolutions, such as life-planning decisions regarding employment, debts, housing, and reproduction. One of the chief interests and concerns that participants have in obtaining a genetic diagnosis actually relates to making informed reproductive decisions, which coincides with concerns that are often examined within the literature (Kole and Faurisson 2010). Faced with the severity of many inherited genetic-based diseases, participants stressed their willingness to be informed as well as provide family members and relatives the chance to inform themselves of their own risks at will. Such impacts on family members have been referred to as “family spillover benefits” (Grosse et al. 2008).

The experiences revealed during the discussions also suggest that the scope of family conflicts and discrimination following the disclosure of a genetic disease may be significant. Family quarrels on the subject of genetic “fault” (i.e. the branch of the family carrying the mutation) have led, in some cases, to the breakdown of family relationships and, ultimately, the isolation of the RD patient (or the paediatric patient’s family). If the disruption of family dynamics is acknowledged as a possible outcome of a RD diagnosis (Buchanan et al. 2013; Bunnik et al. 2014; Foster et al. 2009), the issue remains superficially studied in its social and cultural foundations (e.g. lack of genetic knowledge) and implications (e.g. isolation and stigmatization) and should be given more attention from scholars and health policy makers. Such stakes remain obscure even for the participants if one considers that as mentioned earlier, only a few of those who mentioned family conflicts have framed it openly as a drawback of a genetic diagnosis. Tackling the reason why such family issues are only rarely explicitly considered as a “drawback” by RD patients remains beyond the scope of this study. It would be important that further studies explore this question and analyse whether such conflicts tend to emerge for certain types of RDs more than others.

In short, according to the responses gathered throughout the FGs and the individual interview, the importance ascribed to obtaining a diagnosis by participants from both adult and parental groups is paramount. Even though several participants had poor prognoses (e.g. untreatable and degenerative diseases), mentions of psychological difficulties and effects, such as anxiety or distress, were surprisingly only scarcely pointed out by participants, although frequently mentioned in the literature (Bossuyt and McCaffery 2009). Indeed, rather than being described as a burden, obtaining a diagnosis was depicted as a “deliverance”, allowing for the possibility of grieving, psychological closure, and emotional relief. This echoes studies showing that the quality of life for RD patients is often perceived as “[…] linked more to the quality of care provided, than to the gravity of the illness, or the degree of the associated disabilities” (Kole and Faurisson 2010:247; see also Garrino et al. 2015). There was, notwithstanding, an exception whereby one couple sustained all along that their adult daughter profoundly regretted having searched for a diagnosis and that once a diagnosis was obtained, it essentially bore negative personal effects. According to this couple, having a genetic diagnosis not only brought their daughter no change in terms of medical care but it has limited her reproductive choices.

In sum, our study indicates that obtaining a diagnosis for RD patients may have a *systemic* impact, i.e. affecting medical, familial, psychological, and social dimensions of a person’s life. In addition, it shows how porous the borders delineating such distinct dimensions can be. For instance, the recovery of self-respect vis-à-vis medical professionals (at first glance a personal impact) may have consequences for better access to specialists and follow-up care. Likewise, having access to social and financial support programs may lead to improved care and well-being for the sick patient (for instance, allowing for specialized food and equipment).

### Limitations of the study

Due to acknowledged challenges in recruiting participants suffering from a RD, our study may appear modest in scope. It allowed nevertheless a fine-grained analysis of RD patients’ challenges and experiences, bringing to light new dimensions of the realities of this community. However, the challenges faced within the Canadian public health care system may not be representative of other contexts. The findings are also gender dependent, given that only one male, a father, took part in the study. Further studies in other health care systems and the inclusion of a larger number of male participants may reveal differences in the challenges faced by the RD community found in our study.

## Conclusions

In this study, we explored the impacts of diagnosis from the perspective of RD patients. By delving into the medical, social, and personal mechanisms behind the difficulties experienced by RD patients, our results support the value of a diagnosis and its pivotal role in the lives and health of RD patients.

If “health care providers are poor at knowing what outcomes are important to patients unless they explicitly ask” (Bossuyt and McCaffery 2009:e35), qualitative and in-depth accounts of patients’ experiences may help inform and enrich the parameters for health care providers and decision makers. One of the challenges in assessing the outcomes of diagnosis remains subjective in nature, such that the balance of benefits and drawbacks is likely to vary from individual to individual. Each patient may be affected subjectively and objectively in distinctive ways and to varying extents, according to gender, income, education, and age.

Beyond any individual-related outcome, a diagnosis remains imperative from the perspective of RD epidemiology. A late diagnosis, or even the lack of a diagnosis, often has debilitating and harmful consequences for patients and impairs the body of knowledge on RDs. Practical methods to integrate non-clinical and multidimensional outcomes in health analysis are yet to be developed (Buchanan et al. 2013; Grosse et al. 2009), and the interplay and equilibrium between patient rights and public health system constraints are a dizzying one.

Although social and public funding constraints must not be overlooked, our findings suggest that as long as an official and precise diagnosis is required for patients to be eligible and gain access to basic health, social, and financial services (such as in the Canadian context), diagnostic testing for RD patients should be assessed from a less narrow standpoint than sheer medical or clinical outcomes. A diagnosis is what enables RD patients to make the transition from the chronic and unexplained illnesses with unclear symptoms, and which may not be taken seriously, towards an acknowledgeable and institutionally admitted disease. Furthermore, our study exposes the fluidity and dependency between the diverse orders of effects (personal, social, medical). If the pathways towards health impacts are diverse and indirect, this means that “evaluating the [clinical] management effects alone can never be sufficient for estimating the net health effects of testing” (Bossuyt and McCaffery 2009:e36). Our study thus confirms the importance of non-health outcomes and the necessity of incorporating these factors in diagnostic testing evaluations (Bossuyt and McCaffery 2009; Buchanan et al. 2013; Grosse and Khoury 2006; Halverson et al. 2016).

Overall, our research indicates that RD patients face a series of challenges in their quest for a diagnosis that goes beyond the lack of access to diagnostic technologies, regardless of whether they are traditional genetic testing or NGS. The discordant nature of the doctor-patient relationship that often influences the therapeutic outcome for RD patients presents a greater hurdle. Poor knowledge amongst health care professionals regarding RD and genetics often acts as a bottleneck for a timely diagnosis of RD. Therefore, if new diagnostic technologies such as NGS are to yield their benefits for the RD community, sensitizing primary care physicians to the paradoxical commonality of RD is essential (Anderson et al. 2013; Elliott and Zurynski 2015; Engel et al. 2013; Knight and Senior 2006). In a public health care system, patients still rely on a physician’s referral.

## Footnotes

1 Our study stems from a broader multidisciplinary pan-Canadian project, entitled “CARE for RARE” (C4R), aimed at improving the diagnosis and treatment of rare diseases in Canada, notably through the implementation of NGS as a diagnostic tool.

2 Although it remains beyond the scope of this paper, it is important to note that no official definition of “clinical utility” exists and there are ongoing debates as to its definition (Bossuyt et al. 2012; Bunnik et al. 2014; Grosse and Khoury 2006; Smart 2006). While it is commonly understood as the potential of a test result to inform medical decisions, i.e. the ability of a test to allow for an improvement in health outcomes, such as morbidity or mortality, through clinical decision-making (Bossuyt et al. 2012; Bunnik et al. 2014; Grosse et al. 2010), over the last few years, several authors have put into question the current framework grounded on clinical utility. New notions such as personal utility, multidimensional utility, and extended utility were suggested as ways to yield a more “composite measure of utility” (Foster et al. 2009; see also Buchanan et al. 2013; Bunnik et al. 2014; Grosse and Khoury 2006; Segal 2012). Nevertheless, literature shows that evaluations drawing on extended and wider views of clinical utility are not well established and only few health policies have integrated such frameworks so far (Buchanan et al. 2013; Foster et al. 2009). There is also ongoing discussion about which outcome to include in genetic testing evaluations, a lack of empirical data on different accounts of testing outcomes (Bossuyt et al. 2012; Segal 2012) and of metrics enabling evaluations based on extended notions of utility (Buchanan et al. 2013; Grosse et al. 2009, Grosse et al. 2008; Payne et al. 2007).

3 Questions relating to the practical delivery of genetic testing (Burke and Zimmern 2007), and in particular of NGS (such as how to manage incidental findings), remain beyond the scope of this article.

4 Paediatric disclosure of genetic information is a contentious issue, especially when no treatment is available for the condition. It is also entangled with fundamental notions such as the child’s “best interest” and “autonomy”, which are context-dependent and open to legal interpretation (for an overview, see Geelen et al. 2011; Kleiderman et al. 2013). The distinction between adult and minor patients is decisive when it comes to NGS, given the amount of “incidental findings” that the technique generates. Therefore, our study was designed in order to account for such distinctions (although the analysis of participants’ perceptions on the return of genetic results is beyond the scope of this paper).

5 Given the burden that most parents face when having to take care of a sick child, as well as the precarious conditions of many adult RD patients, the compensation offered was intended to allow interested patients and caregivers to take part in the study without adding extra burdens to their lives and facilitating their travel to FG locations, while covering babysitting costs in case of caregivers.

6 Such discrepancy reflects the fact that some of the parents had two children affected by rare diseases.

7 With regard to NGS technologies such as whole-exome sequencing (WES), it is relevant to note that one of the parents was able to have her child diagnosed using WES after 19 years of searching for a diagnosis. The two undiagnosed cases were in the process of enrolling in research projects to undergo WES (WES being available to patients in Canada only through research projects). Most of the other RDs were diagnosed through traditional genetic tests.

8 Ages of paediatric patients as well as RD types are deliberately omitted to protect patients’ identities. Although we acknowledge that disclosing the RDs would enrich the interpretation of the results by the reader, in doing so, the anonymity of the participants would be put at risk given that they belong to such a small community. Indeed, some of the diseases under discussion affect only a couple of individuals across Canada, making identification a concrete possibility.

9 The French excerpts were translated by the authors. Both English and French excerpts reproduce the spoken language, with minor changes made in order to assure proper grammar, to omit any personally identifiable information (notably, the term “child” was used in the feminine to refer to the particular child in question), and to improve clarity.

10 The claim that a diagnosis has actually made the access to health care more difficult was asserted by different participants of our FGs. The commonality between these participants is that they all suffer from the same RD which renders all connective tissue fragile to trauma. According to these patients’ accounts, doctors are often afraid to take charge of their clinical care due to fears of causing further physical damage. Another case is illustrated below under “life planning and reproductive decision-making.

11 When a conversation is recalled, P indicates each participant.

12 See note 10.

13 For instance, some basic regular check-ups required by particular RD are available in adult hospitals only via the emergency setting (Dogba et al. 2014).

14 For an overview of patients’ perceptions on the importance of obtaining genetic information from NGS testing and the role of the so-called “personal utility”, see Halverson et al. (2016).
